# Mass Spectrometry for Lysine Methylation: Principles, Progress, and Prospects

**DOI:** 10.3390/biomedicines13112825

**Published:** 2025-11-19

**Authors:** Mackenzie G. Cumming, Kyle K. Biggar

**Affiliations:** Institute of Biochemistry, Carleton University, Ottawa, ON K2J0L6, Canada

**Keywords:** lysine methylation, proteomics, mass spectrometry, methyllysine

## Abstract

Lysine methylation is a regulatory post-translational modification with diverse roles across both histone and non-histone proteins. Despite its biological relevance, comprehensive characterization of lysine methylation remains analytically challenging due to its low stoichiometry, subtle mass changes, and the absence of standardized, robust enrichment strategies. Mass spectrometry (MS) has become the cornerstone of methylation analysis, supporting both targeted and proteome-wide investigations. In this review, we examine the evolution of MS-based workflows for lysine methylation, including advances in ionization and fragmentation techniques, high-resolution mass analyzers, and acquisition strategies such as data-independent acquisition (DIA) and parallel accumulation–serial fragmentation (PASEF). We evaluate bottom-up, middle-down, and top-down proteomic approaches and discuss enrichment methods ranging from immunoaffinity and chromatography to chemical derivatization. Particular attention is given to persistent challenges, including proteolytic constraints and isobaric interference, that complicate confident site-level resolution. Finally, we highlight emerging solutions and future directions aimed at improving the sensitivity, specificity, and reproducibility of lysine methylation profiling. Together, this synthesis provides a forward-looking roadmap for optimizing MS workflows in methyllysine proteomics.

## 1. Introduction

Post-translational modifications (PTMs) are central to understanding the regulatory complexity of the proteome. To study these modifications at scale, proteomics has become an essential approach for characterizing protein function, interactions, and regulatory networks. Among PTMs, lysine methylation is particularly intricate, with mono-, di-, and tri-methylation states exerting distinct biological effects and posing specific analytical challenges. Mass spectrometry (MS), the cornerstone of proteomic analysis, has been instrumental in advancing our understanding of lysine methylation and remains the primary method for its detection and characterization. Despite this, our understanding of lysine methylation remains limited, primarily due to its low stoichiometry and the lack of effective enrichment strategies [1,2,3,4,5,6,7,8]. Initial research centered on histones, where lysine methylation serves as a key epigenetic mark that modulates chromatin structure and gene expression. Over the past decade, however, attention has expanded to non-histone substrates, uncovering roles in RNA processing, signal transduction, protein turnover, and the DNA damage response. Lysine methylation is dynamically regulated by lysine methyltransferases (KMTs), which catalyze the addition of methyl groups, and lysine demethylases (KDMs), which reverse them [9]. Dysregulation of lysine methylation and its associated enzymes has been linked to various diseases, including cancer and neurodegeneration, underscoring its potential as both a biomarker and a therapeutic target [10,11,12,13,14,15,16,17,18,19,20].

**Figure 1:**
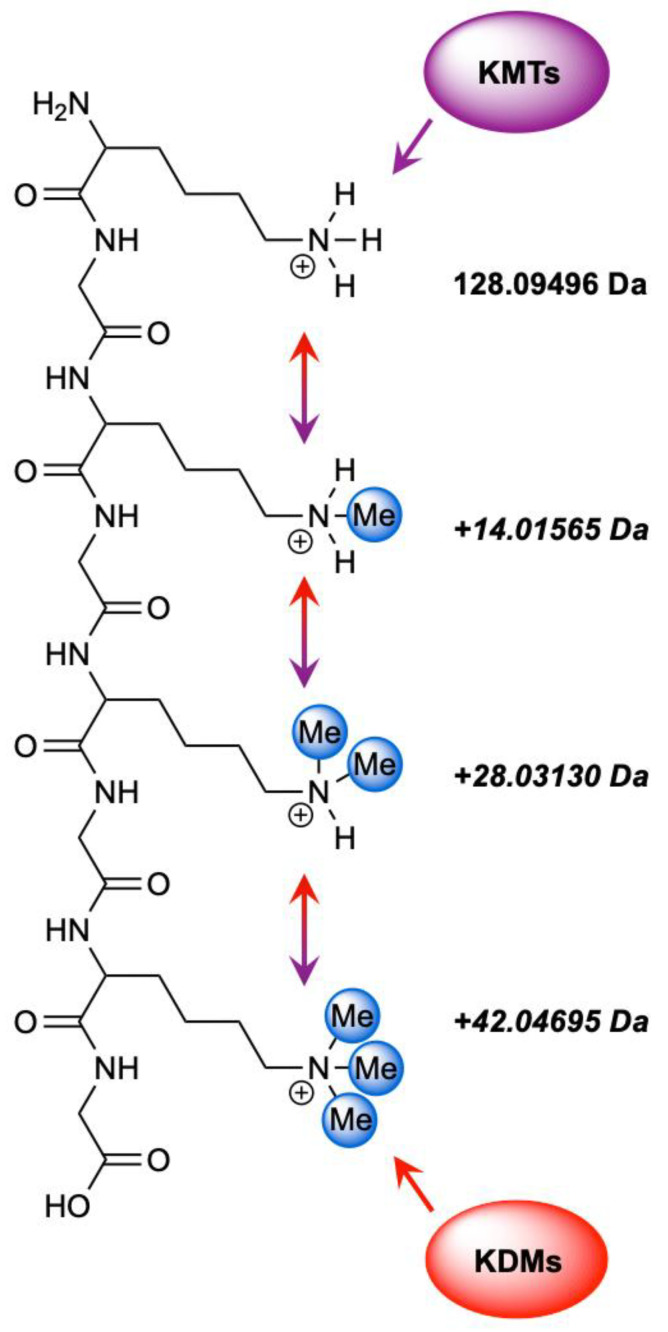
Enzymatic regulation of lysine methylation by KMTs and KDMs. KMTs catalyze the stepwise methylation (purple) of lysine residues to generate mono-, di-, and tri-methylated states (Kme1–3), while KDMs mediate the reverse demethylation process (red). Together, these opposing enzyme families maintain the dynamic balance of lysine methylation across chromatin and other proteins.

Biochemically, lysine methylation is distinct from many other PTMs in that it introduces minimal changes to the residue’s mass and charge. Each methyl group adds only +14 Da without altering the net charge of the lysine side chain (Figure 1). These subtle additions preserve the residue’s physicochemical character yet can markedly influence protein interactions and binding specificity, making enrichment and detection inherently challenging [1,2,3,4,5,6,21,22,23]. Moreover, lysine residues are frequently subject to other PTMs, including acetylation, ubiquitination, and SUMOylation, which may occur concurrently with or in competition with methylation, further complicating site assignment and functional interpretation [24,25]. The minimal mass difference between tri-methylation and acetylation (~0.036 Da) exemplifies how co-occurring or alternative modifications can generate near-isobaric species that are difficult to distinguish, particularly on instruments with lower resolving power. Although distinct in exact mass, these modifications may appear isobaric when their separation approaches the resolving limits of the analyzer. Truly isobaric species, by contrast, share identical masses and cannot be differentiated by mass measurement alone. Additional ambiguity arises from co-occurring modifications and amino acid substitutions, which can obscure identification unless high-accuracy mass measurements and optimized fragmentation schemes are applied. Collectively, such near- and isobaric mass shifts underscore the importance of isotopic labeling, chemical derivatization, orthogonal fragmentation strategies, and complementary computational workflows for confident site localization. Table 1 illustrates how diverse non-methyl modifications and residue substitutions can emulate the incremental mass shifts in lysine methylation, creating isobaric interferences that obscure accurate site and state identification.

The biological consequences of lysine methylation introduce an additional layer of complexity, as each methylation state imparts distinct functional outcomes without major structural perturbations. This context dependence further challenges efforts to interpret site-specific effects and highlights the need for analytical precision when resolving methylation state and stoichiometry.

**Figure 2:**
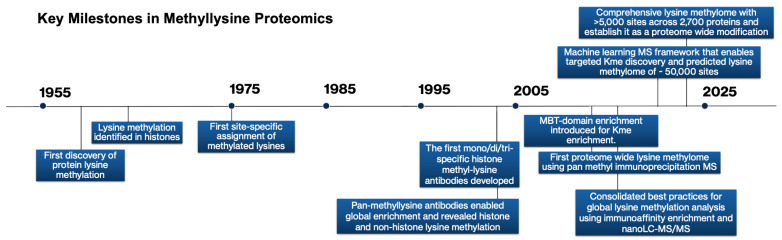
Methodological advances in MS that catalyzed lysine methylation research and expanded proteomic capabilities, highlighting selected pivotal methodological breakthroughs in methylation research.

Although numerous studies have elucidated the biological significance of lysine methylation in chromatin regulation, transcription, and disease, this review focuses on the analytical mass spectrometry strategies that have enabled such discoveries. Prioritizing methodological advances over biological interpretation establishes a comprehensive analytical framework applicable to diverse methylproteomic contexts. The following sections outline the current landscape of MS-based approaches for lysine methylation analysis, emphasizing core methodologies, analytical advantages and limitations, and major areas of application. Particular attention is directed toward emerging developments and prospective research directions. The historical progression of key technological and methodological advances that have shaped lysine methylation research is summarized in Figure 2.

## 2. Foundations of Early Lysine Methylation Discovery

Before the emergence of MS-based proteomics, the detection of lysine methylation relied on a range of biochemical techniques, including Edman degradation, amino acid analysis, radiolabeled methyl donor incorporation, and antibody-based assays such as Western blotting and peptide arrays [6,26,27,28,29]. While these early methods laid much of the groundwork for our understanding of lysine methylation and informed the development of modern detection techniques, they had several limitations, including insufficient specificity and resolution to accurately localize methylation sites or distinguish between mono-, di-, and tri-methylation states. Edman degradation, while capable of detecting methylated lysine with high precision, is inherently low throughput and requires substantial amounts of purified protein, making it impractical for large-scale analyses [6]. Methyl-labeled donor incorporation, such as with radioactively labeled S-adenosyl-L-methionine-dependent (SAM) methyltransferase, enables detection of methylated proteins on a more global scale, but it requires prolonged exposure times and lack the specificity to identify modified residues or to distinguish between potential methylation targets such as arginine, histidine, or terminal amino groups [6,29,30,31]. Immunoblotting with pan-methyllysine antibodies provided a more accessible alternative, but these reagents suffered from poor specificity, variable sensitivity, and lot-to-lot inconsistency [3,32,33,34,35]. The subsequent articulation of the “histone code” hypothesis, which emphasized that specific combinations of histone modifications convey regulatory information [36,37], accelerated the development of modification-specific antibodies that could resolve distinct lysine methylation states [38]. While these reagents represented an important conceptual advance, early versions were still limited by cross-reactivity and inconsistent performance. As with radiolabeling, both pan-specific and modification-specific antibodies lacked site-level resolution. Additionally, these approaches were generally low throughput, offered limited dynamic range for quantification, and often depended on prior assumptions about the modification or target protein. As such, they were not well suited for comprehensive or unbiased analyses of the methylproteome. Ultimately, it has been the technological advancements in MS over the past two decades that have catalyzed lysine methylation research and propelled the field to its current state.

Key Takeaway: Early biochemical methods provided foundational insights but lacked the resolution and scalability required for comprehensive methylation analysis, paving the way for MS-based proteomics.

## 3. Instrumentation Advances in Lysine Methylation MS Workflows

Mass spectrometry (MS) instrumentation has undergone significant evolution, enabling increasingly sensitive and accurate detection of lysine methylation. This section provides a focused overview of those developments most relevant to methylproteomics.

### 3.1. Ionization Techniques

The success of any mass spec workflow begins with effective ionization, and the choice of technique can significantly influence the sensitivity, resolution, and compatibility of downstream analysis [39]. Soft ionization techniques have been fundamental to the rise of proteomics, enabling the ionization of intact peptides and proteins with minimal fragmentation for accurate mass measurement and structural characterization [35,40]. Among these, ESI and MALDI remain the most widely used ionization strategies in proteomics, each offering distinct advantages depending on the analytical context [35,39,40,41,42,43,44,45].

ESI and nanospray ESI (nano-ESI) are well suited for coupling with liquid-phase separation techniques and are compatible with tandem MS, making it ideal for the detection of low-abundance peptides and PTMs in complex biological samples [44,46,47]. MALDI, on the other hand, enables rapid and direct ionization from solid samples, which is advantageous for high-throughput screening or validating known analytes in low-complexity mixtures [41,48]. Because lysine methylation was historically studied primarily in the context of histones, early MALDI-MS methods were rarely applied in methylome discovery, likely due to insufficient resolution for resolving small methylation-related mass shifts and limited sensitivity for detecting short, highly basic and hydrophobic peptides, typical of histone digests [48,49,50,51,52]. However, MALDI in the right context is highly used and efficient in targeted methylation studies of intact proteins within simple, well-defined, and low-complexity analytes [53].

### 3.2. MS/MS Acquisition

Tandem mass spectrometry (MS/MS) serves as the cornerstone of modern proteomics, enabling both targeted and untargeted peptide fragmentation for structural elucidation and site-specific analysis [35,54,55]. By isolating precursor ions and fragmenting them in a predictable manner, MS/MS produces characteristic ion series that reveal peptide sequences and modification sites [35,56]. Such detail is not obtainable from standard MS alone, as the intact peptide mass is often insufficient for unambiguous identification [57,58]. Integration with liquid chromatography, specifically nano-, high-, or ultra-high-performance LC (nLC, HPLC, UHPLC), remains the gold standard, providing improved separation, reduced sample complexity, and enhanced sensitivity for detecting low-abundance peptides. Even with advanced separation techniques, peptide pre-treatment is often necessary prior to LC-MS/MS analysis to enhance detection sensitivity and improve data quality. Strategies for enrichment and sample preparation will be discussed in later sections of this review. In methylation-focused proteomics, LC-MS/MS is most commonly performed on hybrid mass spec platforms, that combine complementary analyzers, such as quadrupoles, ion traps, Orbitraps, or time-of-flight (ToF) systems, to achieve the high resolution, mass accuracy, and scan speed required for confident peptide identification and precise localization of methylation sites [59].

### 3.3. High-Resolution Mass Analyzers

Triple quadrupole (QqQ) and linear ion trap (LIT) instruments have long been used in proteomic methylation studies, though their limited resolving power constrains their performance relative to high-resolution analyzers. QqQ instruments enable sensitive and selective quantification by sequentially filtering precursor and product ions, making them ideal for targeted proteomics [35]. However, their unit-mass resolution restricts the ability to resolve closely spaced or isobaric modifications, limiting discovery-based applications. LIT instruments, which confine ions via oscillating electric fields and support multi-stage fragmentation (MS^n^), provide valuable structural information but are constrained by low ion capacity and space-charge effects that reduce resolution and dynamic range [60,61,62,63]. Advances in ion optics and transmission efficiency have improved their sensitivity and signal stability [64,65], yet these instruments still lack the mass accuracy required for confident discrimination of methylation states and detection limitations remain a challenge [6,35]. These challenges persisted until the early 2000s, when high-resolution mass analyzers and hybrid MS platforms (HR-MS, HR-MS/MS) were introduced alongside advanced fragmentation techniques.

HR-MS platforms revolutionized methylation analysis by providing the mass accuracy necessary to distinguish between mono-, di-, and tri-methylated species and to localize modifications on complex peptide backbones [25,26,66]. Principal HR-MS technologies include Orbitrap, Fourier transform ion cyclotron resonance (FT-ICR), and Q-TOF analyzers, all of which can be coupled to LC-MS/MS configurations, allowing for hybrid platforms. Orbitrap analyzers determine ion mass by measuring oscillation frequencies within an electrostatic field, achieving sub-ppm accuracy and high resolution. Their combination of precision, throughput, and stability has made hybrid Orbitrap platforms the predominant choice for large-scale methylproteomic investigations [6,67]. FT-ICR analyzers employ strong magnetic fields to trap ions and measure cyclotron frequencies with ultra-high resolution, ideal for resolving near-isobaric species in highly complex samples. Q-TOF systems combine quadrupole selection with time-of-flight detection, offering rapid acquisition and accurate-mass capability suitable for both targeted and discovery workflows.

The advent of these high-resolution platforms catalyzed major advances in lysine-methylation proteomics but also introduced trade-offs between resolving power, scan speed, and data complexity. Collectively, they define the analytical boundaries of methylproteomic discovery, enabling discrimination of modification states separated by only a few millidaltons. Comparative performance characteristics of the most widely used analyzers, including their typical operating ranges and trade-offs between resolution and speed, are summarized in Table 2.

### 3.4. Fragmentation

Traditional peptide fragmentation in MS/MS is typically achieved by collision-induced dissociation (CID), which efficiently generates b- and y-type ions and remains well supported by computational frameworks [57,68]. However, the low-mass cut-off and neutral losses inherent to CID can suppress diagnostic ions and compromise PTM localization, particularly for methylated peptides [40,52]. Despite these limitations, CID remains widely used owing to its rapid acquisition speed, sensitivity, and broad compatibility across platforms [59,65].

The introduction of higher-energy collisional dissociation (HCD) on Orbitrap systems addressed several CID-related limitations by using a beam-type collision cell that transmits fragments directly to the Orbitrap analyzer for high-resolution detection [69]. By avoiding the low-mass cut-off, HCD preserves low-*m*/*z* diagnostic ions and enhances methylation-site identification in histone peptides relative to CID [59]. Although overall acquisition is slower due to ion-transfer and accumulation steps required for high-resolution MS/MS detection [70], HCD substantially improved ion-trap performance and has become the dominant fragmentation strategy on hybrid Orbitrap platforms. Like CID, however, HCD remains an ergodic process and can promote over-fragmentation of highly charged, lysine-rich peptides [57,71,72].

On FT-ICR systems, the inefficiency of collisional activation under ultra-high-vacuum magnetic fields limited the utility of CID and HCD, motivating the integration of electron-based dissociation (ExD) methods into high-resolution MS platforms [73,74,75,76,77,78]. Electron capture dissociation (ECD) and electron-transfer dissociation (ETD) induce non-ergodic backbone cleavage while preserving labile modifications making them particularly effective for localization in peptides with multiple methylation sites or higher charge states [79,80]. ETD, implemented on ion-trap and Orbitrap platforms, [73,80,81] is particularly valuable for highly basic histone peptides and multiply methylated substrates [80], though efficiency declines for low charge states (ET-no-D) [82,83,84,85,86]. To mitigate this, activated-ion ETD (AI-ETD) couples ETD with concurrent infrared irradiation to enhance dissociation while maintaining PTM integrity [83]. Building on this foundation, hybrid strategies such as electron-transfer/higher-energy collision dissociation (EThcD) combine non-ergodic and ergodic fragmentation within a single event, generating complementary c/z- and b/y-type ions that improve sequence coverage and site localization while overcoming the limitations of non-hybrid methods [87,88,89,90,91]. EThcD has since become a mainstay in methylproteomics, offering balanced fragmentation for bottom-up, middle-down, and top-down analyses [82,83,87,88,89].

Beyond electron-based strategies, ultraviolet photodissociation (UVPD) provides a complementary high-energy pathway that excites multiple electronic states upon photon absorption, generating rich and diverse ion series with minimal charge-state bias [92,93,94]. The resulting spectra yield near-complete sequence coverage and precise modification-site localization, making UVPD an emerging asset for high-confidence methylproteomic workflows [95,96,97,98].

### 3.5. Modern Data Acquisition and Ion Mobility Enhancements

In parallel with advancements in instrumentation and fragmentation methods, modern data acquisition strategies building on LC-MS/MS have transformed lysine-methylation proteomics by improving sensitivity, coverage, and reproducibility. Proteomic data acquisition is broadly categorized as targeted or untargeted, depending on whether precursor selection is hypothesis-driven or designed for comprehensive discovery [99]. Data-dependent acquisition (DDA) has long served as the standard for discovery-based proteomics and remains prevalent in methylome studies [6,35,100]. However, its reliance on intensity-based precursor selection inherently biases analysis toward abundant species, often missing low-abundance methylated peptides and limiting reproducibility [101,102]. Data-independent acquisition (DIA) overcomes this by fragmenting all ions within predefined *m*/*z* windows, ensuring consistent sampling across runs. Early DIA implementations produced highly multiplexed spectra that were challenging to deconvolute, [103] but the advent of SWATH-MS (Sequential Window Acquisition of All Theoretical Mass Spectra) resolved this by employing narrower sequential windows [104]. SWATH enabled reproducible detection of low-abundance PTMs, establishing DIA as a robust, unbiased alternative to stochastic DDA [105,106]. Since 2015, DIA–SWATH has been increasingly applied in histone-methylation studies for its ability to capture multiple peptidoforms with high quantitative precision [107,108,109]. Untargeted DIA can also detect multiple PTMs in a single experiment, as demonstrated by Robinson et al. (2020), who used a PTM-enriched spectral library to quantify methylation, acetylation, and succinylation concurrently [109].

Complementing untargeted strategies, targeted acquisition approaches such as selected- and multiple-reaction monitoring (SRM/MRM) and parallel reaction monitoring (PRM) are particularly well suited for detecting and quantifying low-abundance peptides owing to their enhanced sensitivity and selectivity [110,111,112] and have shown efficacy for quantifying known methylated peptides. These methods have proven highly effective for validating known methylated peptides. For example, Biggar et al. confirmed 45 of 50 predicted histone-methylation sites using MRM on a triple-quadrupole instrument, while Hoekstra et al. employed high-resolution PRM-MS/MS to confirm 66 histone substrates in a KDM5 demethylation assay [113,114].

To enhance both DDA and DIA, Next-generation acquisition techniques have emerged that further advanced coverage and ion utilization. Parallel Accumulation–Serial Fragmentation (PASEF) integrates trapped ion mobility spectrometry (TIMS) with time-of-flight detection to accelerate sequencing and enhance sensitivity [115]. In histone proteomics, nLC–TIMS–PASEF workflows have improved peptide detection and isobaric resolution while reducing chemical noise through the added ion-mobility dimension [116].

In parallel, ion mobility mass spectrometry (IM-MS) has been increasingly incorporated into DDA and DIA workflows on hybrid instruments to improve precursor selectivity and reduce chemical noise. High-field asymmetric waveform ion mobility spectrometry (FAIMS), widely implemented on Orbitrap platforms, separates ions by differences in mobility under alternating electric fields rather than by collision cross section [117,118,119,120,121], like conventional IM-MS [63]. Introducing an orthogonal separation dimension, FAIMS enhances detection of low-abundance peptides and resolves isobaric PTMs, making it particularly valuable for lysine-methylation profiling in complex sample [119,122,123]. When coupled with nLC–MS/MS, FAIMS recently enabled identification of over 5000 lysine-methylation sites across 2700 human proteins, substantially expanding the characterized methylome [8].

With the analytical capabilities of modern MS platforms firmly established, attention now turns to the proteomic workflows that leverage these technologies to detect and characterize lysine methylation.

Key Takeaway: Advances in ionization, fragmentation, and data acquisition, particularly high-resolution Orbitrap and DIA workflows integrated with FAIMS, have been pivotal for achieving sensitive and site-specific characterization of lysine methylation in both histone and non-histone proteins.

## 4. Proteomic Workflows for Lysine Methylation: Bottom-Up to Top-Down

Quantitative proteomics is commonly categorized into targeted and untargeted mass spectrometry approaches, as introduced earlier. To support these strategies in PTM analyses, three methodological workflows have emerged: bottom-up, middle-down, and top-down proteomics. Each is defined by the extent of proteolysis and the analytical level at which methylation is characterized. These workflows, summarized in Table 3, collectively represent a continuum of analytical resolution from peptides to intact proteoforms. Although often discussed as distinct strategies, they operate in a complementary and iterative framework. Bottom-up proteomics remains the gold standard for discovery-scale analysis due to its scalability and sensitivity, whereas the growing demand for site-specific localization and proteoform context has spurred renewed interest in middle-down and top-down methodologies [75]. Consequently, hybrid workflows that integrate data across these analytical tiers are gaining prominence for achieving comprehensive and confident PTM characterization. An example of an emerging next-generation hybrid approach is discussed at the end of this section.

### 4.1. Bottom-Up Methyllysine Proteomics

Bottom-up proteomics, also known as shotgun proteomics, is the most widely used strategy for analyzing lysine methylation. It involves enzymatic digestion of proteins into peptides, followed by LC-MS/MS analysis, typically on fast, trap-based instruments optimized for high-speed MS/MS scanning [6,35,116,124]. This approach supports both discovery-based and targeted workflows, offering high-throughput data acquisition, extensive proteome coverage, and site-specific localization of PTMs. A typical bottom-up workflow begins with protein extraction, denaturation, reduction, and alkylation to stabilize cysteine residues prior to digestion, most often with trypsin [35,125,126,127]. In recent years, proteases such as Glu-C, Asp-N, Arg-C, and Lys-C have been increasingly integrated into these workflows, either as complements to trypsin or as alternatives [128]. Due to the low stoichiometry of lysine methylation, enrichment methods are commonly employed and traditionally considered necessary to enhance detection sensitivity [8]. However, limited success and a lack of standardization in these approaches have led to their effectiveness being increasingly questioned and reevaluated, as discussed later in this review [7,8]. Data analysis typically involves database searching with variable methylation modifications and strict false discovery rate (FDR) control to ensure confident site assignment. However, because only a subset of peptides from each protein is typically detected, bottom-up analyses can miss modified regions and provide incomplete coverage of methylated proteoforms [129,130,131]. While bottom-up MS provides high sensitivity and throughput, it may obscure combinatorial PTM patterns on intact proteins and often struggles to distinguish between isobaric methylation states.

### 4.2. Top-Down Methyllysine Proteomics

Top-down proteomics analyzes intact proteins without enzymatic digestion, enabling the direct detection and mapping of all modifications in their native context. Although technically more demanding and less sensitive for low-abundance proteins, top-down proteomics has proven particularly powerful for targeted studies of histone variants and other regulatory proteins where combinatorial modifications play a critical role. While traditionally it has faced challenges related to resolution, sensitivity, and intact protein handling, recent improvements in HR-MS/MS have begun to overcome these barriers [132]. Despite these advances, incomplete fragmentation of intact proteins often results in partial sequence coverage and missing fragment ions, which can obscure the precise localization of PTMs [130,131,133]. A typical top-down proteomics workflow involves the isolation of intact proteins, direct ionization by ESI, and fragmentation using ExD techniques, followed by LC-MS/MS analysis for proteoform identification and PTM mapping [134]. Top-down proteomics supports both targeted and untargeted approaches.

### 4.3. Middle-Down Methyllysine Proteomics

Middle-down proteomics is an intermediate strategy between bottom-up and top-down approaches that analyzes large polypeptides rather than fully digested peptides or intact proteins. Enabled by advances in ExD techniques, this approach is applicable to both targeted and untargeted workflows. It largely mirrors bottom-up protocols, but proteins are cleaved into longer peptide segments instead of short tryptic peptides [127]. Generation of these larger polypeptides is achieved through limited proteolysis, wherein enzymes such as Glu-C or Asp-N are operated under sub-stoichiometric and kinetically constrained conditions that favor partial cleavage, thereby preserving extended sequence-context and combinatorial PTM information [135,136,137]. In addition to offering robust quantitative performance comparable to bottom-up proteomics, middle-down provides improved access to combinatorial PTM patterns that are often only captured in top-down analyses [6]. Middle-down proteomics has been particularly valuable in histone research, where conventional digestion often produces overly short, non-informative peptides from histone tails [82,138,139,140]. It is widely regarded as the preferred approach for characterizing combinatorial histone PTMs.

### 4.4. Emerging Hybrid and Next-Gen MS Workflows

The hybrid, or top-‘double-down’, workflow combines two stages of fragmentation with mobility-based separation to improve resolution of isomeric and isobaric proteoforms, particularly in lysine methylation studies [75]. Built on top-down proteomics and high-resolution MS, this approach first applies UVPD followed by trapped ion mobility spectrometry (TIMS), enabling preservation and separation of PTM-containing fragments [82,141,142,143,144]. These are subsequently subjected to mass-selected ECD on a qToF platform equipped with a custom electromagnetostatic (EMS) cell, representing a research-grade configuration that integrates UVPD, TIMS, and electron-based fragmentation within a single system. Although not commercially standardized, this hybrid design exemplifies the direction of next-generation MS innovation, demonstrating how coupling mobility-based and electron-based dissociation enhances PTM localization and proteoform differentiation [76,77,78]. The method successfully resolved Kme1, Kme2, and Kme3 in histone H4 at Lys20 and identified combinatorial acetylation–methylation states, delivering performance comparable to FT-ICR-based ECD with significantly faster acquisition [75]. Notably, its versatility was demonstrated by adapting the workflow to analyze glycosylation on α-synuclein, a non-histone protein, using both a top-down and a middle-down strategy following chymotrypsin digestion [145]. Given its success beyond histones and the growing interest in non-histone lysine methylation, this method may prove valuable for broadening coverage of the methyllysine proteome.

Key Takeaway: Each proteomic workflow—bottom-up, middle-down, and top-down—offers distinct advantages and limitations, and integrating their strengths is key to resolving complex methylation patterns.

## 5. Proteolytic Constraints in Bottom-Up Analysis of Methylated Peptides

In bottom-up proteomics workflows, trypsin is the gold-standard protease due to its highly specific and predictable cleavage pattern at the carboxyl side of lysine and arginine residues [126,146,147,148]. However, the steric hindrance caused by methyl groups, especially in the tri-methylated state, can impair or completely block trypsin cleavage [149]. This leads to missed cleavages, the generation of longer peptide products, and less predictable fragmentation patterns. In addition, many peptides produced by trypsin are naturally short, with nearly one-third expected to fall below the optimal size range for MS/MS analysis, which often results in low-confidence identifications [146,150,151]. The presence of negatively charged amino acids, phosphorylation, or glycosylation near cleavage sites can also interfere with trypsin activity, resulting in incomplete digestion and reduced efficiency [146,148,150].

Tryptic cleavages have been particularly challenging for histone-based analyses, and it is recognized that this approach is largely ineffective due to the high abundance of lysine and arginine residues on histone tails, which leads to overly short, non-informative peptides [113]. To account for this, Garcia et al. applied chemical derivatization using lysine propionylation to block trypsin cleavage at lysine residues, restricting digestion to arginine sites and generating longer, more informative peptides for improved detection and characterization of histone PTMs [152]. First implemented with conventional LC–MS/MS in 2007, this strategy quickly proved highly effective and became a standard in histone proteomics [124,152,153]. By 2009, Garcia and colleagues advanced it into a streamlined “one-pot” workflow, enabling comprehensive and quantitative shotgun profiling of multiple histone PTMs, including lysine methylation [154]. In 2023, this workflow was further refined by Garcia’s group for DIA LC-MS/MS, improving compatibility with modern instrumentation and workflows [155]. Furthermore, continued advances in separation and mass spectrometry technologies have enabled the method to evolve to high-throughput platforms through the integration of nLC-TIMS-ToF instruments [156]. As an alternative approach, middle-down proteomics has been particularly valuable in histone research for characterizing combinatorial histone PTMs [82,138,139,140].

Nonetheless, trypsin remains the protease of choice in most proteomic workflows. Its continued preference is likely driven by its well-characterized cleavage specificity, broad compatibility with existing databases and search algorithms, and the strong MS performance of tryptic peptides. In contrast, while alternative proteases can help address methylation-specific challenges, they often introduce limitations such as unpredictable cleavage patterns and reduced compatibility with standard analysis pipelines [128,146,151,157]. Notably, earlier this year, researchers addressed some of these challenges by providing optimized collision energy (CE) parameters for a range of alternative proteases using CID-based DDA workflows [146]. Their work offers a potential improvement in the identification and analysis of non-tryptic peptides, while highlighting the importance of tailoring MS workflows to the protease used.

Due to the challenges presented by trypsin, there has been growing interest in bottom-up workflows for methylation analyses that incorporate alternative proteases, middle-down workflows, as well as in non-targeted top-down approaches that avoid digestion altogether [7]. These strategies aim to improve sequence coverage, site localization, and detection of methylation sites that may be obscured by inefficient tryptic cleavage.

Key Takeaway: The limitations of trypsin digestion in methylation studies have driven interest in alternative proteases and hybrid workflows that improve peptide coverage and modification detection.

## 6. Enrichment Strategies for Methylated Peptides

Bottom-up proteomics coupled with LC-MS/MS has been the primary analytical platform supporting advances in lysine methylation research. Within this framework, enrichment strategies are often critical for increasing the detectability of methylated peptides in protein digests, which are typically low in abundance and exhibit sub-stoichiometric modification levels. While enrichment methods have played a transformative role in the study of many PTMs and have traditionally been considered essential for lysine methylation analysis, a broadly accepted and standardized enrichment strategy has not yet been established [1,2,3,4,5,6,7,8]. This lack of standardization remains a major limitation in efforts to achieve comprehensive and confident profiling of the lysine methylome [1,2,3,4,5,6,21,22,23]. To date, the most significant progress in this field can largely be attributed to advances in MS sensitivity, rather than to improvements in enrichment techniques. In response to this limitation, several enrichment strategies have been explored, including immunoaffinity enrichment, affinity chromatography, and chemical derivatization [3,8,23,32,158,159]. Although enrichment has traditionally been regarded as a critical step for enhancing detection sensitivity, ongoing improvements in MS performance have contributed to a growing trend toward workflows that bypass enrichment entirely in favor of direct analysis [8].

Broadly, enrichment methods can be divided into antibody- or reader domain-based approaches, which rely on biological recognition of methyllysine, and antibody-free approaches, which exploit the physicochemical or chemical properties of methylated peptides. Despite their utility, these reagents suffer from limitations such as sequence-context bias and inconsistent specificity, which restrict their ability to capture the full diversity of methylated peptides. Antibody-free methods such as chromatography and chemical derivatization provide complementary advantages, offering reproducibility, scalability, and reduced bias that can augment antibody-based strategies [67,100].

### 6.1. Biological Recognition-Based Enrichment

Immunoaffinity enrichment is the most widely used strategy for isolating lysine-methylated peptides and proteins prior to LC-MS/MS analysis. This approach typically employs pan-methyllysine antibodies that recognize specific methylation states independently of the surrounding amino acid sequence. Despite their utility, these reagents often display sequence bias, variable specificity, and lot-to-lot inconsistency, limiting their reliability for comprehensive methylome profiling [4,8,160,161]. To improve specificity, natural methyllysine reader domains, such as malignant brain tumor (MBT) and chromodomains (CDs), have been repurposed as affinity reagents [2,23,113,114,162,163]. Most recently, the plant homeodomain 1 (PHD1) of KDM5B has become of particular interest [8,114,164]. These domains are frequently used in targeted approaches and offer the added advantage of including negative controls using binding-deficient mutants, which help discriminate true methylation-dependent interactions from nonspecific background [8]. Nonetheless, enrichment at the peptide level has yielded modest datasets relative to other PTMs, and efforts to improve sensitivity have included strategies such as combining enrichment with affinity chromatography or derivatization-based approaches.

### 6.2. Affinity Chromatography

Chromatography-based strategies offer an antibody-independent approach to enrich or fractionate methylated peptides, capitalizing on their distinct physicochemical properties. Methylated peptides, particularly those with methylated lysine or arginine, tend to be highly hydrophilic and carry multiple positive charges due to missed cleavages at methylation sites during tryptic digestion. Techniques such as strong cation exchange (SCX), hydrophilic interaction liquid chromatography (HILIC), and isoelectric focusing (IEF) have been evaluated for their ability to enrich these peptides. Among them, HILIC has demonstrated superior performance, whereas SCX and IEF often suffer from co-elution of histidine-rich or miscleaved peptides [2,127,165]. Nevertheless, high-pH SCX has shown promise in improving selectivity by minimizing histidine interference and reducing miscleavages through the use of multiple proteases [160]. When combined with reversed-phase LC-MS/MS, this method has enabled the identification of hundreds of methylation sites using minimal starting material. While chromatography-based enrichment lacks the sequence specificity of immunoaffinity methods, it offers higher reproducibility, lower cost, and improved scalability. Other innovations, including charge-suppression chemistry and metabolic labeling with allyl-SAM, further enhance the specificity and resolution of methylproteome profiling through chromatography-based workflows [166,167].

### 6.3. Chemical Derivatization

Chemical derivatization offers a powerful alternative and complement to traditional enrichment strategies for lysine methylation, providing improved specificity and the ability to target specific methylation states. While this tunability is a major advantage, it often requires extensive chemical optimization, limiting its accessibility. The tertiary amine coupling by oxidation (TACO) method [67,159], for example, selectively oxidizes dimethyllysine to reactive aldehydes, which can be derivatized with affinity tags for enrichment and downstream MS analysis. This approach enables profiling of dimethyllysine sites, including those poorly recognized by antibodies, and has been applied in single-molecule sequencing workflows.

Chemo-enzymatic labeling using the SAM analog ProSeAM, biosynthetically generated from ProSeMet in live cells, allows endogenous methyltransferases to install propargyl groups on methylated lysine, arginine, or histidine residues. These can then be enriched via click chemistry for site-specific, in vivo methylome mapping [67]. Together, these strategies, among others, expand the detectable methylome and provide complementary tools for high-resolution analysis of protein methylation in complex systems. Following enrichment, the next critical step in lysine methylation analysis is the accurate and confident assignment of modification sites from MS/MS spectra.

Key Takeaway: Despite advances in MS sensitivity, enrichment remains a critical and unresolved bottleneck in methylproteomics due to a lack of standardized and broadly effective approaches.

## 7. Quantitative Labeling Strategies for Lysine Methylation

A major challenge in profiling methylation events at the substrate- and site-specific levels using LC–MS/MS is high false discovery rates (FDRs) due to common isobaric interference. To address this limitation, labeling-based orthogonal validation strategies are used to confirm true in vivo methylation and reduce false positives, including hM-SILAC and TMT labeling [2,4,22,161,168]. Although many methylation studies have employed label-free quantification (LFQ) for discovery-scale analyses, labeling approaches such as SILAC and TMT have been especially influential in enhancing confidence for low-stoichiometry PTMs like lysine methylation.

Heavy methyl stable isotope labeling by amino acids in cell culture (hM-SILAC) is a metabolic labeling strategy used to validate lysine methylation by incorporating isotopically labeled methyl groups, typically via ^13^CD3-methionine, into proteins during cell growth [169,170,171]. These heavy methyl groups are transferred through endogenous SAM metabolism, resulting in in vivo labeled methylation events that can be distinguished from non-enzymatic or artifactual modifications by MS. The incorporation of isotopically labeled methyl groups produces a characteristic +4 Da mass shift per methylation event, directly linking the modification to enzymatic activity and enhancing confidence in methyl site assignment [5,20,172,173]. In the ProMetheusDB study, hM-SILAC was applied to support large-scale profiling of the human methylproteome [172]. By pairing light and heavy methylated peptides at the MS1 level, the authors reduced false positives and generated a high-confidence dataset containing both canonical and novel methylation sites. To enable automated analysis, they developed hmSEEKER 2.0, a machine learning algorithm optimized for hM-SILAC data, which facilitated the construction of ProMetheusDB, one of the most comprehensive resources of experimentally validated methylation sites. Recognizing the limited multiplexing capacity and cell culture dependency of SILAC, the authors proposed isobaric mass tags (i.e., TMT labeling) as a more scalable alternative for high-throughput methylation studies, which is an alternative method used for orthogonal validation.

Isobaric mass tags such as iTRAQ and TMT were introduced to enable multiplexed quantitative proteomics and allow simultaneous analysis of multiple samples in a single LC–MS/MS run [174,175,176]. As high-resolution Orbitrap platforms became widely adopted, TMT emerged as the dominant isobaric labeling strategy due to its improved reporter ion performance and reproducibility, making it especially powerful for quantifying low-abundance PTMs, including lysine methylation. The main limitation of TMT labeling is ratio compression caused by co-isolation interference, which disproportionately affects low-abundance peptides and presents a critical challenge for PTM studies where modification occupancy is often low. This limitation was largely mitigated by the development of SPS–MS^3^ workflows [177], later applied to histone PTM profiling to enable more accurate quantification of lysine methylation states [178]. To further improve sensitivity, recent studies have introduced isobaric trigger channels, which boost the ion intensity of target peptides and improve their identification and quantification [179,180,181]. Earlier this year, this strategy was applied to lysine methylation analysis, where TMT labeling combined with a KDM-enriched trigger channel enabled simultaneous quantification of over 300 methylation sites and the majority of KDMs across multiple breast cancer cell lines [7]. These findings revealed cell line-specific methylation patterns and suggested that antibody-based enrichment may not be essential for deep methylome coverage.

An emerging application of isobaric labeling strategies is single-cell proteomics (SCP), which adapts TMT workflows to enable proteome analysis at single-cell resolution. The SCoPE-MS method pioneered the use of TMT labeling with carrier channels, enabling the quantification of hundreds of single cells across multiple 8-plex sets and substantially improving sensitivity [182]. Subsequent optimization in SCoPE2 introduced narrow isolation windows, apex-targeted ion sampling, and data-driven optimization (DO-MS) and identification (DART-ID), greatly enhancing quantitative accuracy and throughput [183]. Parallel advances in ion transmission efficiency, acquisition speed, and sensitivity have further strengthened label-free single-cell workflows [184,185,186,187,188]. While initial applications targeted global protein quantification, more recent work demonstrates feasibility for PTM detection at the single-cell level [189]. Applying single-cell proteomics to lysine methylation promises to uncover cell-to-cell heterogeneity in methylation dynamics that bulk measurements cannot resolve, marking a frontier for quantitative PTM analysis [189]. In parallel, advances in high-sensitivity label-free approaches such as diaPASEF on the timsTOF SCP have demonstrated that robust quantification of PTMs can also be achieved without isobaric tags, even at the single-cell level [189,190]. Nonetheless, isobaric labeling remains the predominant strategy for orthogonal validation in lysine methylation studies.

Accurate site localization and confident annotation of lysine methylation events remain among the most difficult bioinformatics challenges in PTM analysis. The subtle mass differences between methylation states and near-isobaric modifications such as acetylation or formylation can lead to high false discovery rates (FDRs) if not carefully controlled during database searching. Reliable localization depends on both high-resolution MS/MS spectra and diagnostic fragment ions that distinguish true methylation from co-occurring PTMs. In addition, spectral library coverage for methylated peptides remains limited, restricting automated validation and quantification across studies. Efforts such as hmSEEKER 2.0, developed for hM-SILAC datasets, and recent machine learning-based scoring algorithms are improving methyl site confidence by integrating isotopic labeling, retention time prediction, and ion intensity features. Expanding and standardizing spectral libraries dedicated to lysine methylation will be essential to improve cross-laboratory reproducibility and enable broader biological interpretation of methylproteomic datasets.

Key Takeaway: Orthogonal validation techniques such as hM-SILAC and TMT labeling enhance confidence in site-specific methylation identification and reduce false discovery rates.

## 8. Conclusions

Lysine methylation is a functionally diverse and analytically challenging PTM that continues to reveal its importance in both histone and non-histone regulatory pathways. MS has been central to progress in this field, enabling the detection, localization, and quantification of methylation events across increasingly complex biological systems. However, despite substantial technological advancements, confident and comprehensive analysis of the lysine methylome remains hindered by several key factors: low modification stoichiometry, subtle mass differences between methylation states and isobaric modifications, the absence of standardized enrichment strategies, and the proteolytic limitations of conventional bottom-up workflows. Recent developments in instrumentation, data acquisition strategies, and fragmentation methods have significantly improved the resolution, sensitivity, and reproducibility of methylation analyses. The growing use of middle-down and top-down proteomics, and hybrid workflows incorporating ion mobility and advanced dissociation methods, suggests a shift toward more integrative strategies capable of resolving complex PTM patterns at the proteoform level. Establishing a standardized quantification strategy will be critical for addressing the analytical challenges posed by lysine methylation. Looking ahead, future efforts should focus on refining enrichment reagents, optimizing proteolytic workflows catered to lysine methylation, and exploring alternative MS workflows. Furthermore, expanded spectral libraries and improvements in bioinformatic tools for site validation will be essential to fully characterize the lysine methylome and its functional roles. As the field continues to evolve, these integrated approaches will be key to translating lysine methylation from a mechanistic hallmark into a robust analytical target for systems biology and disease research.

## Figures and Tables

**Table 1:** Representative PTMs and amino acid substitutions that generate near- or isobaric mass shifts relative to lysine methylation. Examples shown include biologically relevant PTMs and sequence-level variants that may confound methylation assignment in proteins.

Modification Type	Alteration	Observed Mass Shift (Da)	Spectral Ambiguity	Deviation from Target PTM Mass (Da)
Acetylation	K→KAc	+42.0106	Kme3 mimic	0.03635 Da
Carbamylation	K→Kcarb	+43.0058	Kme3 mimic	0.95885 Da
Formylation	K→Kfo	+27.9949	Kme2 mimic	0.0364 Da
Amino Acid Substitution	Val→Thr	+15.9949	Kme1 mimic	1.97925 Da
	Ala→Ser	+15.9949	Kme1 mimic	1.97925 Da
	Phe→Tyr	+15.9949	Kme1 mimic	1.97925 Da
	Lys→Arg	+28.0070	Kme2 mimic	0.02430 Da
	Cys→Met	+28.0313	Kme2 mimic	0→identical
	Ala→Val	+28.0313	Kme2 mimic	0→identical
Deamidation	Glu→Gln	−0.9840	Misleading spectrum	0.9840 Da
	Asp→Asn	+0.9840	Misleading spectrum	0.9840 Da

**Table 2:** Comparative performance characteristics of common mass analyzers used in lysine methylation proteomics. Reported values, applications, and limitations represent approximate performance ranges under typical proteomics acquisition conditions. Reported metrics are instrument- and mode-dependent; higher resolving power can be achieved in specialized scan modes, although these are rarely employed in LC–MS/MS owing to trade-offs between resolution and efficiency.

Instrument Class	Mass Analyzer	Mass Range (*m*/*z*)	Resolution	ΔMass at 100 *m*/*z* (Da)	Mass Accuracy	Applications	Key Limitations
HIGH	FT-ICR	50–10,000	>5 M	0.00002	<1 ppm	High-confidence distinction of Kme states in intact proteins.	High cost; Slow acquisition
	Orbitrap	50–6000	140–500 K	0.0002	<1 ppm	Accurate distinction of Kme states.	Lower resolving power compared with FT-ICR
	Q-TOF	50–40,000	20–80 K	0.001	<5 ppm	Rapid analysis with moderate resolution for lysine methylation states.	Limited resolution for near-isobaric Kme states
LOW	Ion Trap	50–2000	5–25 K	0.004	5–50 ppm	Routine peptide sequencing and structural characterization.	Inability to resolve isobaric lysine methylation states; Moderate mass accuracy
	QqQ	50–4000	1–5 K	0.02	>100 ppm	High-throughput targeted quantification.	Inability to resolve isobaric Kme states; Poor mass accuracy

**Table 3:** Comparative overview of standardized mass spectrometry workflows for lysine methylation proteomics. Bottom-up, middle-down, and top-down strategies provide complementary views of the methylproteome, differing primarily in the extent of proteolysis, fragmentation mode, and analytical resolution. Bottom-up approaches dominate discovery-scale analyses owing to their depth and quantitative scalability, whereas middle-down workflows capture combinatorial histone and domain-level PTM patterns. Top-down proteomics uniquely resolves intact proteoforms, though with lower throughput and greater computational complexity. Together, these workflows constitute a continuum of analytical resolution for dissecting lysine methylation across peptides, domains, and intact proteins.

Workflow	Bottom-Up	Middle-Down	Top-Down
Resolution	Site-level	Domain-level	Proteoform-level
Analyte	Peptide (5–30 aa)	Polypeptide (25–90 aa)	Intact protein (>100 aa)
Proteolysis	Full (trypsin, Lys-C)	Partial/Limited (Glu-C, Asp-N)	None
Fragmentation	CID, HCD, ETD, EThcD, AI-ETD	ETD, AI-ETD, EThcD, UVPD	ECD, ETD, AI-ETD, UVPD
Enrichment Strategies	Immunoaffinity (pan- or state-specific antibodies); reader domain affinity; chromatographic separation (SCX, HILIC, IEF); chemical derivatization	Chromatographic or charge-based fractionation (SCX, HILIC); chemical derivatization; occasional antibody or reader-domain pull-downs	Rare; protein-level immunoprecipitation or fractionation (e.g., IEF)
Quantification	Discovery: LFQ, SILAC, hM-SILAC, TMT/iTRAQ Targeted: PRM, SRM/MRM	Discovery: LFQ, SILAC, hM-SILAC, TMT/iTRAQ Targeted: PRM, targeted DIA	Discovery: LFQ Targeted: PRM (rare)
Applications	Global methylome profiling; site-specific quantification of lysine methylation; comparative analysis across conditions	Mapping of combinatorial histone modifications; domain-level analysis of PTM crosstalk; profiling structured protein regions	Proteoform-resolved methylation analysis; characterization of intact isoforms and variant-specific methylation states
Strengths	High analytical depth and throughput; established informatics for FDR control and site localization	Resolves combinatorial PTM states and histone tail variants	Enables direct proteoform mapping and distinction of isobaric or isomeric PTMs
Limitations	Loss of proteoform context; missed cleavages; limited ability to resolve combinatorial PTMs	Lower coverage and throughput; complex fragmentation spectra	Incomplete fragmentation, low dynamic range, and high computational demand

## Data Availability

No new data were created or analyzed in this study.
